# Development of a novel multi-epitope vaccine for brucellosis prevention

**DOI:** 10.1016/j.heliyon.2024.e34721

**Published:** 2024-07-18

**Authors:** Kaiyu Shang, Yuejie Zhu, Tingting Tian, Huidong Shi, Zhengwei Yin, Yueyue He, Juan Shi, Jianbing Ding, Fengbo Zhang

**Affiliations:** a State Key Laboratory of Pathogenesis, Prevention and Treatment of High Incidence Diseases in Central Asia, The First Affiliated Hospital of Xinjiang Medical University, Urumqi, Xinjiang, 830011, PR China; b Reproductive Medicine Center, The First Affiliated Hospital of Xinjiang Medical University, Urumqi, Xinjiang, 830011, PR China; c Department of Immunology, School of Basic Medical Sciences, Xinjiang Medical University, Urumqi, Xinjiang, 830011, PR China

**Keywords:** *Brucella*, Multi-epitope vaccine, Epitope prediction, Molecular modeling, Molecular docking

## Abstract

Brucellosis, a zoonotic disease caused by *Brucella*, presents a significant threat to both animal and human health. In animals, the disease can lead to infertility, miscarriage, and high fever, while in humans, symptoms may include recurrent fever, fatigue, sweating, hepatosplenomegaly, and joint and muscle pain following infection. Treatment often involves long-term antibiotic therapy, placing a substantial psychological and financial burden on patients. While vaccination is crucial for prevention, current animal vaccines have drawbacks such as residual virulence, and a safe and effective human vaccine is lacking. Hence, the development of a vaccine for brucellosis is imperative. In this study, we utilized bioinformatics methods to design a multi-epitope vaccine targeting *Brucella*. Targeting Heme transporter BhuA and polysaccharide export protein, we identified antigenic epitopes, including six cytotoxic T lymphocyte (CTL) dominant epitopes, six helper T lymphocyte (HTL) dominant epitopes, one conformation B cell dominant epitope, and three linear B cell dominant epitopes. By linking these epitopes with appropriate linkers and incorporating a Toll-like receptor (TLR) agonist (human beta-defensin-2) and an auxiliary peptide (Pan HLA-DR epitopes), we constructed the multi-epitope vaccine (MEV). The MEV demonstrated high antigenicity, non-toxicity, non-allergenicity, non-human homology, stability, and solubility. Molecular docking analysis and molecular dynamics simulations confirmed the interaction and stability of the MEV with receptors (MHCI, MHCII, TLR4). Codon optimization and in silico cloning validated the translation efficiency and successful expression of MEV in *Escherichia coli*. Immunological simulations further demonstrated the efficacy of MEV in inducing robust immune responses. In conclusion, our findings suggest that the engineered MEVs have the potential to stimulate both humoral and cellular immune responses, offering valuable insights for the future development of safe and efficient *Brucella* vaccines.

## Highlights

• Our selection of two proteins as candidates for a *Brucella* multi-epitope vaccine.
• Two candidate proteins are associated with intracellular infection and virulence in *Brucella*.
• Constructed multi-epitope vaccines are candidates for *Brucella* vaccines.

## Graphical abstract

**Figure:**
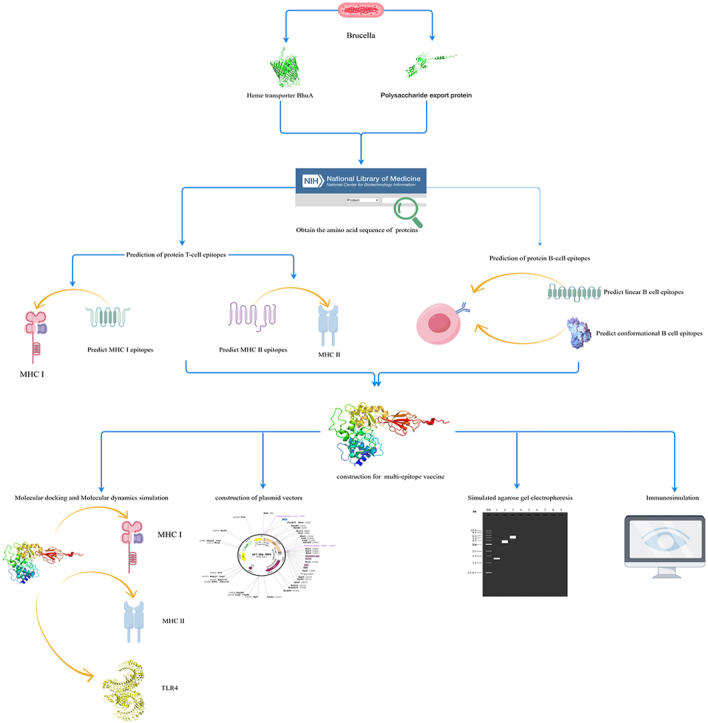

## 1 Introduction

Brucellosis, a zoonotic disease caused by *Brucella* bacteria, is characterized by its ability to survive and multiply within host macrophages [1]. Afflicted animals commonly exhibit symptoms such as infertility, abortion, and reduced milk production, resulting in significant economic losses for the livestock industry [2]. Human infection typically occurs through direct contact with infected animals or consumption of contaminated dairy products, leading to clinical manifestations like intermittent fever, arthritis, orchitis, hepatosplenomegaly, and depression [3]. Although rarely fatal, brucellosis can cause severe debilitation and disability [4]. Recent research indicates a global annual incidence of 1.6 million to 2.1 million new cases, significantly higher than the previously estimated 500,000 cases [5,6], posing substantial threats to public health. Treatment usually involves a combination of antibiotics, but prolonged use may lead to side effects and the development of drug-resistant strains, presenting significant therapeutic challenges [7]. Vaccination remains the most effective preventive measure against *Brucella* infection. The current *Brucella* vaccines for animals consist of live attenuated vaccines, including S19, Rev.1, S2, SR82, and RB51 [8,9]. Among these, RB51 is the only officially approved attenuated vaccine. While these vaccines provide good immune protection in animals, there is a risk of residual virulence that could potentially lead to human infection and abortion complications in pregnant cows [10]. Regrettably, there is currently no effective vaccine available for brucellosis in humans. With the advancement of bioinformatics and a deeper understanding of the pathogenic mechanisms of *Brucella*, there is anticipation for new vaccines to replace traditional live attenuated vaccines in controlling brucellosis. The multi-epitope vaccine, a subunit vaccine [11], utilizes the reverse vaccinology method to predict potential candidate vaccine proteins from the entire genome sequence [12]. Bioinformatics analysis tools are then employed to predict and analyze the epitopes of these candidate proteins. By identifying dominant epitopes that can elicit effective cellular immunity and humoral immune responses for inclusion in vaccine construction, and integrating other bioinformatics technology evaluations, a novel multi-epitope vaccine is developed. This approach not only saves experimentalists time and resources but also holds promise for improved vaccine efficacy [13].This study utilized bioinformatics techniques to develop a multi-epitope vaccine against *Brucella*, focusing on T cell epitopes and B cell epitopes of Heme transporter BhuA and polysaccharide export protein. To assess the feasibility of this vaccine construction, various bioinformatics software tools were employed to analyze the interaction between the developed vaccine and host immune receptors. The findings indicated that the constructed vaccine is a promising and safe candidate, potentially serving as a foundation for the prevention and treatment of brucellosis.

## 2 Materials and methods

### 2.1 Selection of target proteins

Heme transporter BhuA and polysaccharide export protein of *Brucella* were chosen as target proteins for our study. When *Brucella* resides within host macrophages for extended periods, it utilizes the Heme transporter BhuA to absorb heme as a source of iron [14]. The bacteria's ability to acquire sufficient iron is crucial for its survival within the host organism. Therefore, the Heme transporter BhuA plays a significant role in *Brucella*'s intracellular infection. Polysaccharide export proteins are responsible for the transmembrane export of polysaccharides, which are associated with bacterial virulence and mechanisms of drug resistance [15]. Hence, polysaccharide export proteins are vital in the process of *Brucella* causing harm to the host organism. We utilized the ProtParam tool (https://web.expasy.org/protparam/) to analyze the physical and chemical properties of the target proteins, such as the amino acid count, molecular formula, isoelectric point (PI) value, instability index, and grand average of hydropathicity (GRAVY). Furthermore, we employed VaxiJen 2.0 [[16], [17], [18]](http://www.ddg-pharmfac.net/vaxijen/VaxiJen/VaxiJen.html) to assess the antigenicity of the target protein, setting a threshold of 0.4 [19]. The VaxiJen 2.0 server predicts protective antigens based on the physicochemical properties of proteins, using an antigenicity threshold of 0.4. Additionally, we conducted sensitivity analysis of the target proteins using AllergenFP v.1.0 [20,21] (https://ddg-pharmfac.net/AllergenFP/). Lastly, VirulentPred2.0 [22] (https://bioinfo.icgeb.res.in/virulent2/predict.html) was utilized to determine whether the target protein is a virulence factor of *Brucella*. Virulence proteins play a crucial role in the pathogenesis of pathogens by facilitating invasion, survival, and multiplication within the host, as well as enhancing their pathogenicity and evading host defense mechanisms. The identification of these factors, particularly as potential targets for vaccines or drugs, is essential for research in vaccine and drug development. Therefore, we utilized VirulentPred2.0 to identify virulence proteins.

### 2.2 Obtain the amino acid sequence of the target proteins

The amino acid sequences of *Brucella* Heme transporter BhuA and polysaccharide export protein were obtained from the NCBI database (https://www.ncbi.nlm.nih.gov/).

### 2.3 Prediction of protein signal peptides

The signal peptide, consisting of 5–30 hydrophobic amino acids at the N-terminus of a protein, serves to direct newly synthesized proteins to specific subcellular locations. Since we are using an E. coli system to express the multi-epitope vaccine, the signal peptide is unnecessary and can be eliminated. We employ both the SignalP 6.0 server [23](https://services.healthtech.dtu.dk/services/SignalP-6.0/) and LiPOP1.0 [24] (https://services.healthtech.dtu.dk/services/LipoP-1.0/) to forecast protein signal peptides.

### 2.4 Prediction of protein epitopes

An epitope is a distinct chemical group within an antigen molecule that plays a crucial role in dictating the specificity of the immune response. This region is where the antigen binds specifically to the antigen receptor (TCR/BCR). Epitopes are categorized into T cell epitopes and B cell epitopes based on the specific recognition by T and B cells.

#### 2.4.1 Prediction of protein T-cell epitopes

T cell epitopes are linear epitopes processed by antigen-presenting cells (APC) and bound to major histocompatibility complex (MHC) molecules for presentation on the APC surface. CD4^+^ T cells recognize antigen peptides presented by MHCII molecules, while CD8^+^ T cells recognize peptides presented by MHCI molecules. This categorizes T cell epitopes into cytotoxic T lymphocyte (CTL) epitopes and helper T lymphocyte (HTL) epitopes. Given *Brucella*'s nature as an intracellular parasitic bacterium, the identification of T cell epitopes is crucial for understanding its pathogenicity and potential immune responses. In this study, we focused on high-frequency alleles HLA-A*1101 (13.46 %), HLA-A*0201 (12.50 %), and HLA-A*0301 (10.10 %) for CTL epitope prediction, and HLA-DRB1*0701 (16.35 %), HLA-DRB1*1501 (8.65 %), and HLA-DRB1*0301 (7.69 %) for HTL epitope prediction in Xinjiang, China [25,26].The CTL epitope of the target protein is predicted using IEDB (http://tools.immuneepitope.org/) and the NetCTLpan1 server [27,28] (https://services.healthtech.dtu.dk/service.php?NetCTLpan-1.1) for CTL epitope prediction. Three alleles of HLA-A are selected with a length of 9mers, as most HLA molecules have a strong preference for binding 9mers, while other default thresholds remain unchanged.In addition, we utilized IEDB and NetMHC-IIpan-4.0 [29](https://services.healthtech.dtu.dk/service.php?NetMHCIIpan-4.0) to predict the HTL epitope of the target protein, adjusting for the fact that NetCTL-pan1.1 counts from 0 by adding 1 to the sequence. Specifically, we chose three HLA-DRB1 alleles with a length of 15mers for predicting HTL epitopes, while keeping other thresholds at default settings. Subsequently, we employed VaxiJen 2.0, AllergenFP v.1.0, ToxinPred [30](https://webs.iiitd.edu.in/raghava/toxinpred/design.php), and NCBI online tools to assess the antigenicity, sensitization, toxicity, and conservation of T cell epitopes. Through this comprehensive analysis, we identified the top three overlapping sequences that exhibited strong immunogenicity, high antigenicity, non-allergenicity, non-toxicity, and conservation, ultimately selecting them as T cell dominant epitopes. Notably, our epitope screening process did not take into account the signal peptide sequence of the target protein.

#### 2.4.2 Prediction of protein B-cell epitopes

B cell epitopes are specific antigen surface regions that antibodies recognize, bind to, and trigger an immune response in humoral immunity. There are two types of B cell epitopes: linear epitopes and conformational epitopes. Linear B cell epitopes(LBE) of proteins are predicted using SVMtrip [31], IEDB, and ABCpred Prediction Server. These online servers are widely used for predicting protein linear B cell epitopes, with overlapping sequences from at least two prediction results being considered as linear B cell dominant epitopes. This approach significantly enhances the reliability of epitope prediction.We utilized IEDB's Ellipro tool [32] to predict conformational B cell epitopes (CBE), which are formed through protein folding that brings distant residues into proximity. The default threshold value was maintained throughout the analysis. Subsequently, we employed VaxiJen 2.0, AllergenFP v.1.0, ToxinPred, and NCBI to assess the antigenicity, sensitization, toxicity, and conservation of the identified B cell epitopes. Our final selection criteria included sequences with strong immunogenicity, high antigenicity, non-allergenicity, non-toxicity, and conservation, leading to the identification of B cell dominant epitopes. Notably, the signal peptide sequence of the target protein was not considered during epitope screening.

### 2.5 Vaccine construction for MEV

During the multi-epitope vaccine design phase, linkers are employed to bridge dominant epitopes together. Given the relatively low immunogenicity of multi-epitope vaccines, TLR agonists and auxiliary peptides are attached to the N-terminus of the epitopes to enhance immune responses. Specifically, human beta-defensin-2 [33] (hBD2) was selected as a TLR(toll like receptor) agonist and connected to the Pan HLA-DR epitopes (PADRE) sequence using an EAAK linker to elicit CD4^+^ T cells [34], thereby enhancing the effectiveness of the multi-epitope vaccines [35]. The PADRE sequence is then linked to the CTL epitope using a GGGS linker, known for its superior stability compared to AAY [36]. Additionally, the HTL epitope is connected with GPGPG, the B cell epitope with KK, and a polyhistidine tag is added at the C-terminus for the purification and identification of the vaccine post-expression [[37], [38], [39], [40]]. Table 1 shows the detailed structure and role of MEV.

**Table 1:** Detailed structure and role of MEV.

Structure	Sequence	Role
Human-Beta-defensin-2(hBD2)	MRVLYLLFSFLFIFLMPLPGVFGGIGDPVTCLKSGAICHPVFCPRRYKQIGTCGLPGTKCCKKP	Adjuvant and TLR agonist, stimulate immune response
Pan HLA-DR epitopes (PADRE)	AKFVAAWTLKAAA	Adjuvant, enhancing the immunogenicity of multi-epitope vaccine
Linker	EAAK, GGGS, GPGPG	Preventing the generation of junctional epitopes (neoepitopes) and enhancing the folding, stability of MEV
CTL epitopes	SVNGTLSYK	Effectively activate CD8^+^ T cells, thus effectively killing cells infected by Brucella
	RTNGNMILK	
	NVIDPAFLK	
	YLRDPDVSV	
	YVPGMTVQK	
	AAAGGFSPR	
HTL epitopes	KDNIEATGGTVLTYK	effectively activate CD4^+^ T cells and participate in cellular and humoral immunity
	NDRRYRKFNTQQVGA	
	SQKLGNDPEEYRSRG	
	AGQYSYVPGMTVQKA	
	AAGQYSYVPGMTVQK	
	GQYSYVPGMTVQKAI	
B cell epitopes	DIGYERSRDKADRLIKMNMG	Activate B cells and promote differentiation of B cells into plasma cells to produce specific antibodies
	DNIEATGGTVLTYKDIEKLQ	
	G126,E127,V128,G129, A130,A131,G132,I146, A147,A148,A149,G150, G151,F152,S153,P154, R155,A156,N157,Q158, E159,R191,E192,R193, L194,F195	
Polyhistidine tag	HHHHHH	Facilitate purification and identification of multi-epitope vaccine

### 2.6 We predicted the physical and chemical properties, antigenicity, sensitization, solubility, toxicity and non-homology of MEV

The physical and chemical properties of MEV were predicted using the ProtParam tool, including molecular formula, number of amino acids, PI value, instability index, and grand average of hydropathicity (GRAVY). MEV antigenicity was predicted using VaxiJen 2.0 with a threshold of 0.4. Sensitization of MEVs was assessed using AllergenFP v.1.0 to ensure they are not sensitizing, as sensitizing vaccines can lead to cross-reactions. SOLpro was utilized to predict the protein solubility tendency of MEV after overexpression in E. coli, achieving an overall accuracy of 74.15 % with a threshold of 0.5. ToxinPred was employed to determine the potential toxicity of MEV. BLASTP provided by NCBI was used to compare MEV with the entire human proteome to identify any human homology. It is crucial for MEV not to exhibit human homology, as homologous proteins can trigger autoimmune reactions. A protein with an e value of less than 0.005 and ≤ 35 % homology is considered a non-human homologue [41].

### 2.7 We predicted the secondary and tertiary structure of MEV

The secondary structure of MEV was predicted using SOPMA online analysis software [42] (http://www.ibcp.fr/predict.html), while the tertiary structure was predicted using RoseTTAFold's online site Robetta (https://robetta.bakerlab.org/). RoseTTAFold utilizes deep learning techniques to efficiently and precisely predict protein structures with limited data [43,44].

### 2.8 Quality assessment and refinement of tertiary structures

The tertiary structure of MEV predicted by Robetta was assessed for quality using Procheck [45] in UCLA-DOE LAB-SAVES v6.0, a tool designed for evaluating protein structures. Specifically, the Ramachandran Plot was utilized to analyze the geometric conformation of amino acid residues, assessing the psi and phi dihedral angles in the protein structure. This analysis helps identify any unreasonable amino acid conformations, thus evaluating the overall rationality of the structure. Subsequently, GalaxyRefine [46] was employed to refine the tertiary structure, with Procheck used once again to assess the quality of the refined structure. GalaxyRefine reconstructs all side chain conformations and iteratively relaxes the structure through short molecular dynamics simulations following side chain repacking perturbations, ultimately enhancing both global and local structure quality.

### 2.9 Molecular docking

The binding affinity of MEV to immune cell receptors (MHC-I, MHC-II, and TLR-4) was investigated through molecular docking. The HDOCK server [[47], [48], [49], [50], [51]] (http://hdock.phys.hust.edu.cn/) was utilized for docking the refined MEV with the mentioned immune cell receptors. The HDOCK server employs a hybrid docking algorithm that combines template modeling and free docking to predict their interactions automatically. The tertiary structures of these receptors were sourced from the Protein Data Bank (PDB) with ID numbers 1I1Y, 1KG0, and 4G8A [52],respectively. The best model was selected from the top ten models based on docking scores, and the docking results were visualized using PyMOL. Subsequently, the interaction between the receptor and the ligand, along with its two-dimensional image, was analyzed using Ligplot + v.2.2 software [53].

### 2.10 Molecular dynamics simulation

We used Gromacs 2022.3 [54,55] to conduct molecular dynamics simulations of TLR4 protein and the TLR4 complex binding to MEV (TLR4-MEV) to evaluate the impact of TLR4 protein binding to MEV on its protein structure and stability. The steps for molecular dynamics simulation of TLR4 protein are as follows: The simulations were performed under static conditions at 300K and 1 Bar pressure, employing the Amber99sb-ildn force field and Tip3p water model for the solvent. The system's total charge was neutralized by the addition of Na + ions. The simulation protocol included energy minimization using the steepest descent method, followed by 100,000 steps of NVT and NPT equilibration with a coupling constant of 0.1 ps and a duration of 100 ps. Subsequently, a free molecular dynamics simulation was conducted for a total of 5,000,0000 steps with a step size of 2 fs, totaling 100 ns. The molecular dynamics simulation steps of TLR4-MEV are as follows: The MEV molecule underwent GAFF force field preprocessing with AmberTools22, followed by hydrogenation and RESP potential calculations using Gaussian 16W. The resulting potential data was integrated into the system topology file. The subsequent steps are the same as the molecular dynamics simulation steps of TLR4 protein. Post-simulation analysis involved the use of internal tools for trajectory analysis, including calculation of Root Mean Square Deviation (RMSD), Root Mean Square Fluctuation (RMSF), Radius of Gyration (Rg), Hydrogen Bond (HBond) and binding free energy (ΔG_MMGBSA_) for each amino acid movement trajectory.

### 2.11 Codon adaptation and in silico molecular cloning

To facilitate successful cloning, we optimized the vaccine constructs by using the online codon optimization tool ExpOptimizer. The codons of MEV were optimized for analysis and optimization, with E. coli chosen as the expression host. Two restriction endonuclease sites (XHOI and *Bam*HI) were excluded, and the DNA sequence of MEV was obtained from the results. The quality of codon optimization was assessed using the codon adaptation index (CAI) and GC content, with a CAI score of 1 and a GC content of 30%–70 % considered optimal [56]. For cloning MEV, the vector pET-28a(+) was selected. Subsequently, the DNA sequence of MEV was imported into SnapGene7.0.2 for primer design. The principles of primer design included a primer length between 15 and 30 bp, a Tm value of 60 °C, and a GC content between 40 % and 60 %. Following completion of the primer design, a *Xho*I restriction site (CTCGAG) was added before the 5′ end of the forward primer, with a protective base (CCG) included to allow proper enzyme function. Similarly, a *Bam*HI restriction site (GGATCC) was added before the 5′ end of the reverse primer, along with a protective base (CGC) to ensure enzyme activity. Subsequently, the DNA sequence of MEV was amplified using SnapGene7.0.2 software. Analysis of the multiple cloning site (MCS) region of pET-28a (+) revealed suitable *Xho*I and *Bam*HI restriction endonuclease sites, into which the amplified MEV target gene sequence was inserted to complete the in vivo cloning process.

### 2.12 Simulate agarose gel electrophoresis of MEV

Agarose gel electrophoresis was simulated for the target gene (post-PCR), pET-28a (+) vector, and recombinant plasmid using SnapGene7.0.2 software. The experiment was conducted in TBE buffer with a 1 % concentration.

#### 2.12.1 Immunosimulation

The immune response of the designed MEV was evaluated using C-ImmSim [57], a tool that simulates three compartments simultaneously to represent different anatomical regions in mammals: bone marrow, thymus, and lymph nodes [57,58]. The simulation parameters included a random seed of 12345, a simulation volume of 50, and a simulation step of 1050. The host HLA selection consisted of Xinjiang high-frequency alleles HLA-A*1101, HLA-A*0201, HLA-B*5101, HLA-B*3501, HLA-DRB1*0701, and HLA-DRB1*1501 [25], with injections administered three times at time steps 1, 84, and 168.

## 3 Results

### 3.1 Target protein selection

The target proteins analyzed were the *Brucella* Heme transporter BhuA and the polysaccharide export protein. According to ProtParam, the molecular weight of Heme transporter BhuA is 72932.84 Da, with a theoretical pI value of 5.71, 143 amino acid residues, and an instability index (II) of 31.12. This value falls below the critical threshold of 40, indicating that Heme transporter BhuA is a stable protein. Additionally, it has an average hydrophilicity coefficient of −0.486, suggesting it is a hydrophilic protein. On the other hand, the polysaccharide export protein has a molecular weight of 21186.27 Da, a theoretical pI value of 9.27, and 36 amino acid residues. Its instability index (II) is 33.67, still below the critical value of 40, making it a stable protein. The average hydrophilicity coefficient for this protein is −0.031, indicating it is also hydrophilic.In VaxiJen 2.0, the antigenicity prediction results for Heme transporter BhuA and polysaccharide export protein were 0.6018 and 0.5500, respectively, both exceeding the threshold of 0.4, suggesting strong antigenicity. In AllergenFP v.1.0, the antigenicity prediction results for Heme transporter BhuA and polysaccharide export protein indicate a sensitization result of “possibly not sensitizing”. In VirulentPred2.0, the prediction results for Heme transporter BhuA and polysaccharide export protein are labeled as 'virulent', suggesting that these proteins are virulence factors of *Brucella*.

### 3.2 Target protein sequence search

The GenBank number of the Heme transporter BhuA is AIJ88289.1, and the corresponding amino acid sequence is as follows:

MKFTRMLVLASTSLLATVATSQAQEVKRDTKKQGEVVLKPITIISHGKDNIEATGGTVLTYKDIEKLQPANVSELFSRQSSIAVSGGGGPSKRIHVLGMEQSNLAVSVDGVPQTATSWHHTGSNVIDPAFLKRVEVEAGAAAADSGFGAAAGAIRYETVNALDLLEPGKTFGARIIGSYGTNGRGFSGSTAAYGLKDGFDWLLMLHGTSGHNYKNGDGTEILGTEPAARNILGKAGYEFDGNRIDIGYERSRDKADRLIKMNMGLPGDTEYPLEVARDSVNIKYTRTDATDMWDPEVQFYYNRNDYWRNDYQNRTNGNMILKEDLYGGKLQNTFTIDYGKITAGIDFGKHDYNTDNYGHNDRRYRKFNTQQVGAFTQGRFEFDNGFSLSTGARYDYSRFADWNDEVFSDSGASVNGTLSYKFNEHIEVFAGASRTWLGYVLGDYGYVHARNNAFYTDPTFSPGRARNYKAGVNFGGADWSAGITLFDTRIAGLPNYDSQKLGNDPEEYRSRGFTLNARYIWNYTTIGATFTKAKVTAGDDPVLPNSGSFMPIGDMATLFIDQEIPDYNMKVGATLAWAGRISDEAATAANFYDQPAYTVVNAYAEWNPPAVKNMTLRVGVENLFNENYYERTSFAPSQNRGGIDAVWAPGRTFTFQTAFKF.

The GenBank number of the polysaccharide export protein is UVW32680.1, and the corresponding amino acid sequence is as follows:

MATTIQSKRKSHIIFLALGFAAMTLAGCASYRPAPPAFHEALNQPYLLDAGDRVRITVFEQPSLSNNYSVDQSGYIAFPLIGSVPARGKTSKQLESIIASKLRGGYLRDPDVSVEVDRYRPIFVMGEVGAAGQYSYVPGMTVQKAIAAAGGFSPRANQENVDITRQFNGEVLTGRVLISSPILPGDTLYVRERLF.

### 3.3 Prediction of signal peptides

**Fig. 1:**
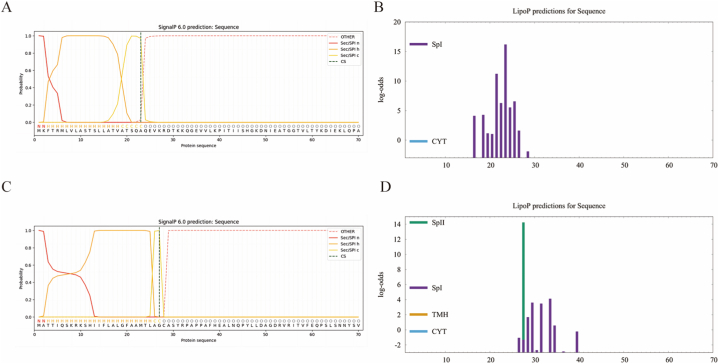
Protein signal peptide prediction results. (A)Predicted Heme transporter BhuA signal peptide results using SignalP 6.0.(B)Predicted Heme transporter BhuA signal peptide results using LiPOP1.0. (C)Predicted polysaccharide export protein signal peptide results using SignalP 6.0.(D)Predicted polysaccharide export protein signal peptide results using LiPOP1.0.

Signal peptides of the proteins Heme transporter BhuA and polysaccharide export protein were predicted using SignalP6.0 and LiPOP1.0. The results from both prediction tools were in agreement. The signal peptide sequence of Heme transporter BhuA is MKFTRMLVLASTSLLATVATSQAQ, while the signal peptide of polysaccharide export protein is MATTIQSKRKSHIIFLALGFAAMTLAGC (Fig. 1[A-D]).

### 3.4 Results of epitope prediction

#### 3.4.1 T-cell dominant epitopes

The CTL epitopes of the target protein were predicted using IEDB and NetCTLpan1, while the HTL epitopes were predicted using IEDB and NetMHC-IIpan-4.0. We identified the top 15 overlapping epitopes with higher scores from the two predicted websites. Subsequently, VaxiJen 2.0, AllergenFP v.1.0, ToxinPred, and NCBI were utilized to analyze the antigenicity, sensitization, toxicity, and conservation of these epitopes. The top 3 overlapping sequences with higher scores were selected as T cell dominant epitopes. Ultimately, six CTL dominant epitopes and six HTL dominant epitopes were identified. These epitopes exhibit high immunogenicity, antigenicity, non-allergenicity, non-toxicity, and conserved sequences (Table 2).

**Table 2:** Selected epitopes of multi-epitope vaccines.

Protein	Epitope types	Results of epitope prediction	immunogenicity	Antigenicity	allergenicity	toxicity	conservativity
Heme transporter BhuA	CTL epitopes	SVNGTLSYK	0.9789	1.6721	non-sensitizing	non-toxic	conservative
		RTNGNMILK	0.9777	0.7112			
		NVIDPAFLK	0.9425	1.0455			
	HTL epitopes	KDNIEATGGTVLTYK	0.8811	1.1063			
		NDRRYRKFNTQQVGA	0.8459	0.8419			
		SQKLGNDPEEYRSRG	0.8224	0.8727			
	LBE	DIGYERSRDKADRLIKMNMG	1.0000	0.6228			
		DNIEATGGTVLTYKDIEKLQ	0.8550	0.6587			
polysaccharide export protein	CTL epitopes	YLRDPDVSV	0.8917	1.4716			
		YVPGMTVQK	0.5913	1.2399			
		AAAGGFSPR	0.4773	1.4483			
	HTL epitopes	AGQYSYVPGMTVQKA	0.9450	0.8746			
		AAGQYSYVPGMTVQK	0.9364	0.9579			
		GQYSYVPGMTVQKAI	0.8135	0.7132			
	LBE	DVSVEVDRYRPIFVMGEV	0.8000	0.6102			
	CBE	G126, E127, V128, G129, A130, A131, G132, I146, A147, A148, A149, G150, G151, F152, S153, P154, R155, A156, N157, Q158, E159, R191, E192, R193, L194, F195	0.7430	0.6022			

CTL: cytotoxic T lymphocyte.

HTL: Helper T lymphocyte.

LBE: linear B cell epitopes.

CBE: conformational B cell epitopes.

#### 3.4.2 B-cell dominant epitopes

Through bioinformatics analysis, we identified 1 conformational B cell epitope and 3 linear B cell epitopes. These epitopes exhibit strong immunogenicity, high antigenicity, high conservation, and are non-sensitizing and non-toxic (Table 2).

### 3.5 The construction of MEV

**Fig. 2:**
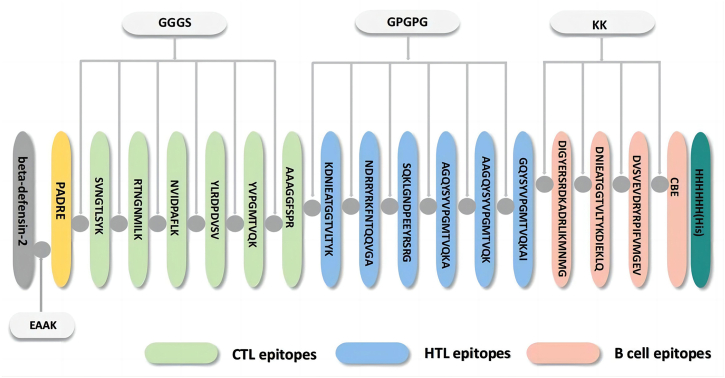
Epitope connection.

The epitope connection sequence of MEV is shown in Fig. 2.

### 3.6 Physicochemical properties, antigenicity, sensitization, solubility, toxicity and non-homogeneity of MEV

The physical and chemical properties of MEV were predicted using the ProtParam tool. The analysis revealed that MEV has a molecular formula of C1959H3090N560O573S17, a molecular weight of 44200.51 Da, a theoretical pI value of 9.72, and consists of 421 amino acids. The instability index (II) of MEV was found to be 28.42, below the stability threshold of 40, indicating that MEV is a stable protein. Additionally, the GRAVY value of −0.380 suggests that MEV is a hydrophilic protein. The antigenicity of MEV, predicted using VaxiJen2.0, was 1.0085, surpassing the threshold of 0.4, indicating good antigenicity. AllergenFPv.1.0 predicted MEV as 'possibly not sensitizing', suggesting no cross-reactivity in vivo. The solubility of MEV, assessed by SOLpro, was 0.511481, exceeding the threshold of 0.5. ToxinPred analysis indicated that MEV is non-toxic. BLASTP analysis in NCBI showed that MEV has minimal homology with the human proteome, with an e value much less than 0.005, confirming it as a non-human homologous protein. Overall, the design of MEV appears to be feasible.

### 3.7 Prediction of MEV secondary structure and tertiary structure

**Fig. 3:**
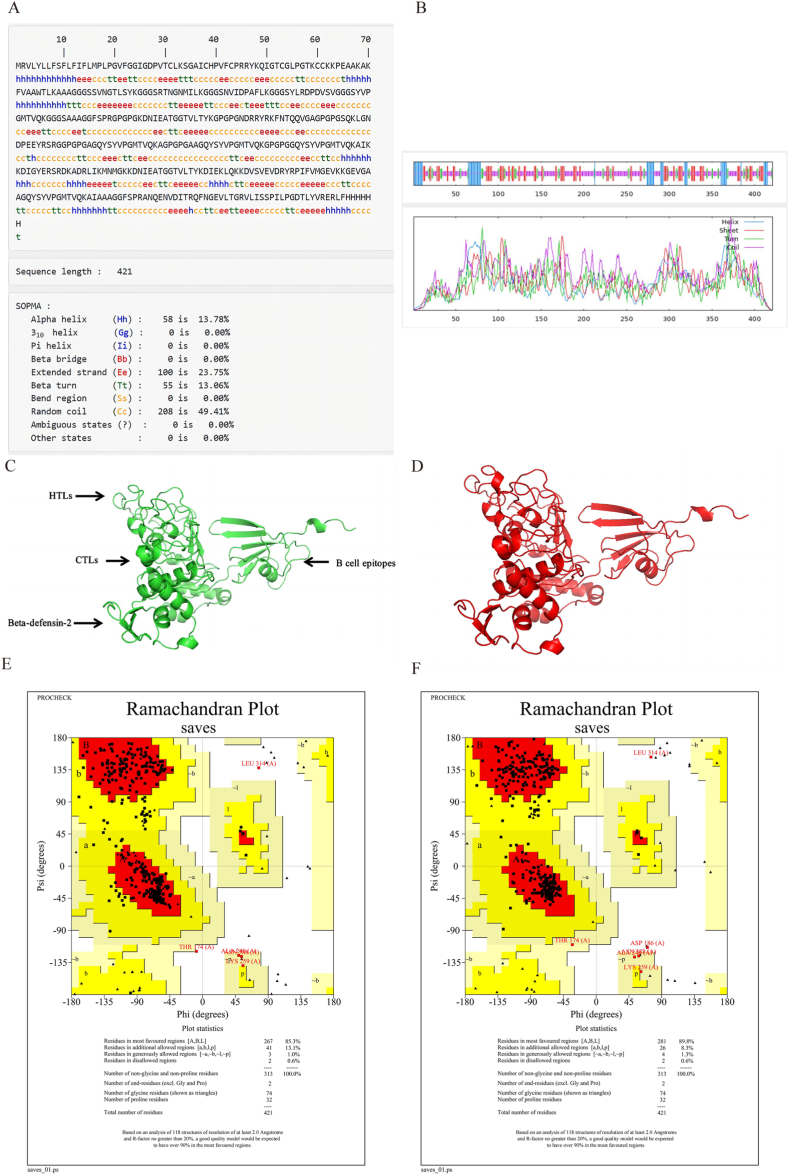
Prediction results and quality assessment results of MEV. (A and B) Prediction results of MEV secondary structure. (C)Prediction results of MEV tertiary structure. (D)MEV three-level structure refinement results. (E)Ramachandran Plot of MEV tertiary structure.(F)Ramachandran Plot after the refinement of the three-level structure of MEV.

The secondary structure of MEV was predicted using SOPMA, revealing that α-helices accounted for 13.78 %, β-turns for 13.06 %, random coils for 49.41 %, and extended chains for 23.75 % (Fig. 3A and B). The tertiary structure of MEV was constructed using RoseTTAFold's online platform Robetta, selecting model1, and visualized in PyMOL (Fig. 3C). The consistency between the predicted secondary structure and the constructed tertiary structure confirms the overall validity of the tertiary structure prediction.

### 3.8 Quality assessment and refinement of three-level structures

Procheck conducted a quality assessment of the tertiary structure of MEV. The Ramachandran Plot results revealed that 85.3 % of the residues were in the most accepted region, while 13.1 % were in the additional allowed region (Fig. 3E). Subsequently, GalaxyRefine was utilized to refine the tertiary structure of MEV (Fig. 3D), followed by a quality evaluation using Procheck. The defined Ramachandran Plot indicated that 89.8 % of the residues were in the most accepted region, with 8.3 % in the additional allowed region (Fig. 3F). These findings suggest that refining the tertiary structure of MEV using GalaxyRefine leads to an improvement in structural quality.

### 3.9 Molecular docking results

**Fig. 4:**
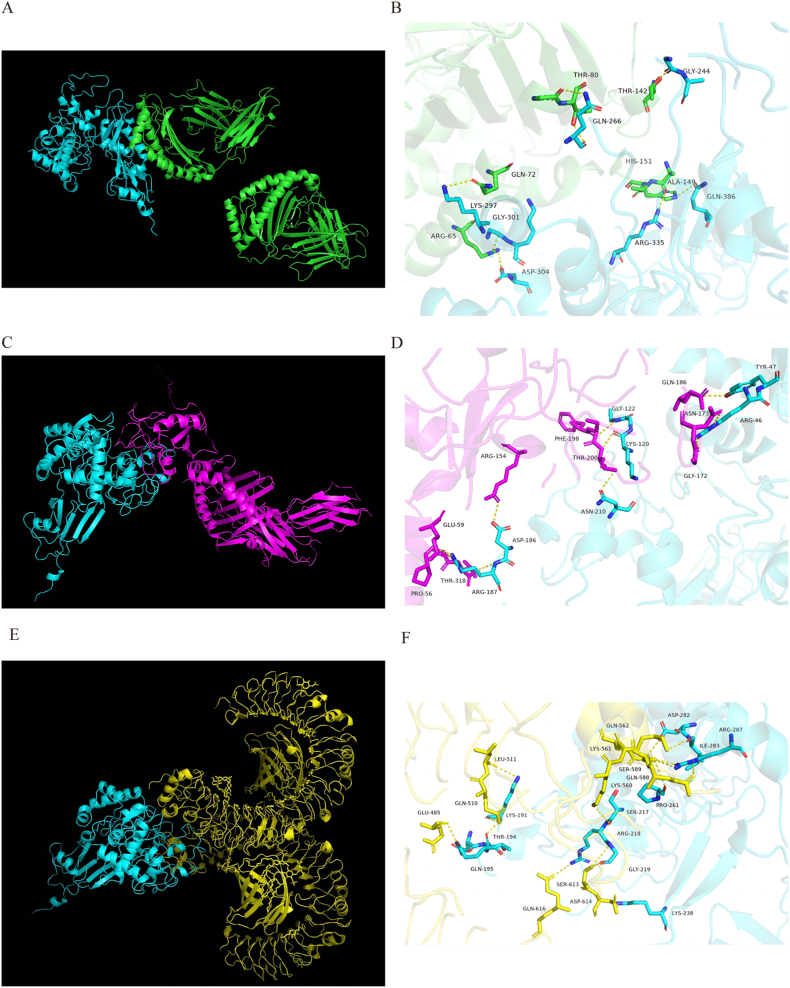
PyMOL display of molecular docking results. (A) Structural diagram of the MEV-MHCI complex drawn using PyMOL: cyan is MEV and green is MHCI. (B) Analysis of the interaction of MEV-MHCI complex and its three-dimensional image using PyMOL. (C) Structural diagram of the MEV-MHHCII complex drawn using PyMOL: cyan is MEV and magenta is MHCII. (D) Analysis of the interaction of MEV-MHCII complex and its three-dimensional image using PyMOL. (E) Structural diagram of the MEV-TLR4 complex drawn using PyMOL: cyan is MEV, yellow is TLR4. (F) PyMOL is used to analyze the interaction of the MEV-TLR4 complex and its three-dimensional image.

**Fig. 5:**
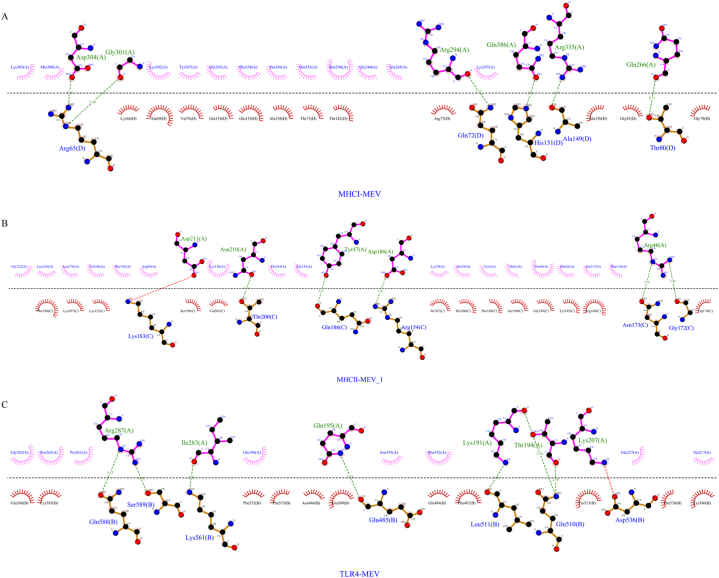
Two-dimensional interaction interface predicted by Ligplot + v.2.2 (A) Analysis of the interaction of MHCI-MEV complex and its 2D image using Ligplot + v.2.2. (B) Analysis of the interaction of MHCII-MEV complex and its 2D image using Ligplot + v.2.2. (C) Analysis of the interaction of TLR4-MEV complex and its 2D image using Ligplot + v.2.2.

Through the HDOCK server, we obtained the molecular docking results of the top ten models. Selecting the model with the most negative docking score for further analysis, we focused on the interactions between receptors (MHCI, MHCII, TLR4) and ligands (MEV). The results revealed that the docking score between MHCI and MEV was −287.13, with a ligand RMSD of 78.65 Å and a confidence level of 0.9395. Similarly, the docking score between MHCII and MEV was −321.53, with a ligand RMSD of 111.40 Å and a confidence level of 0.9686. Lastly, the docking score between TLR4 and MEV was −330.59, with a ligand RMSD of 55.23 Å and a confidence level of 0.9737. Visualization of the docking structures and three-dimensional interactions was performed using PyMOL (Fig. 4A–C, E and Fig. 4B–D, F, respectively). Analysis indicated the formation of 8 hydrogen bonds between MHCI and MEV, 10 hydrogen bonds between MHCII and MEV, and 15 hydrogen bonds between TLR4 and MEV. Ligplot + v.2.2 was utilized to predict a two-dimensional interaction interface, where red dotted lines represent salt bridges and green dotted lines represent hydrogen bonds (Fig. 5[A-C]).

### 3.10 Molecular dynamics simulation

**Fig. 6:**
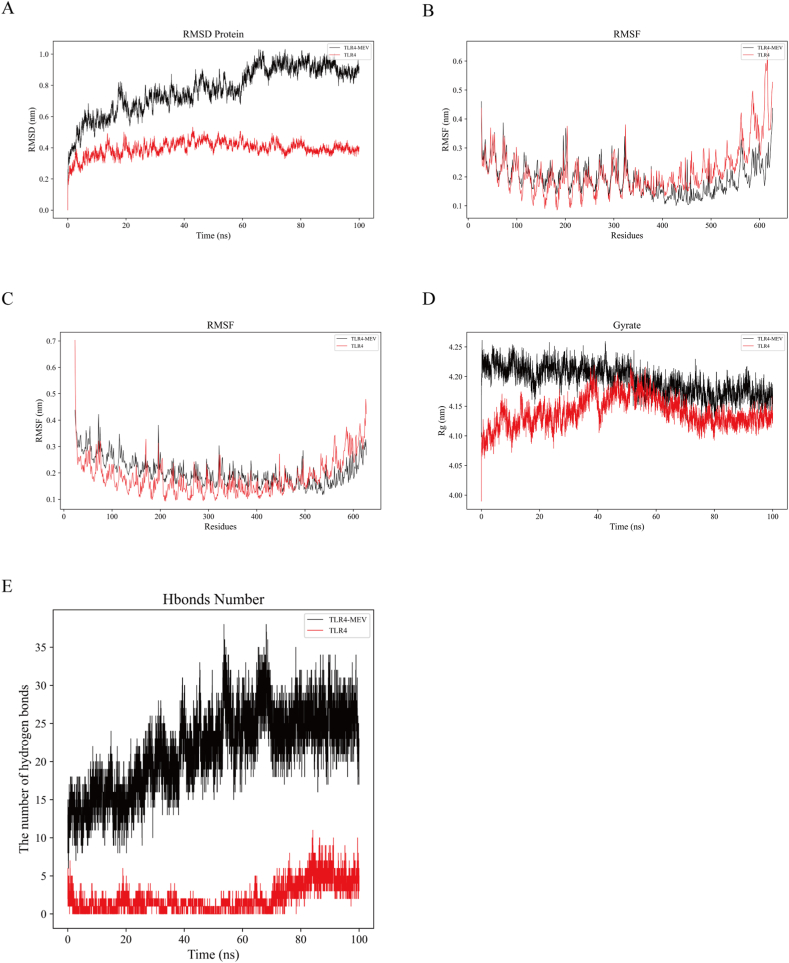
Molecular dynamics results (A) RMSD diagram of TLR4 and TLR4-MEV (B) RMSF diagram of TLR4 protein A chain and MEV binding to TLR4 A chain (C) RMSF diagram of TLR4 protein B chain and MEV binding to TLR4 B chain (D) Rg diagram of the TLR4 and TLR4-MEV (E) HBond diagram of the TLR4 and TLR4-MEV.

To analyze the impact of MEV binding on TLR4 protein stability, molecular dynamics simulations were conducted using the GROMACS tool. RMSD is a metric that evaluates changes in protein structure. In this simulation, we calculated the RMSD of TLR4 protein and TLR4-MEV complex and plotted the RMSD plot (Fig. 6A). The results showed that the RMSD of TLR4 protein and TLR4-MEV complex gradually tended to equilibrium after 70 ns (Fig. 6A), indicating that the binding between MEV and TLR4 was stable. RMSF is the average of atomic position changes over time, which can characterize the flexibility and intensity of movement of protein amino acids throughout the simulation process and determine the applicability of ligand-protein interactions at simulation time. Amino acids located further from the active site will exhibit higher RMSF values. Fig. 6B shows the changes in RMSF values when TLR4 protein A chain and MEV combine with TLR4 A chain. Fig. 6C shows the changes in RMSF values when TLR4 protein B chain and MEV combine with TLR4 B chain. Overall, their The RMSF value changes in a similar trend, roughly fluctuating between 0.15 and 0.25 nm, and the overall structure of the complex is relatively stable. Rg is a measure of the overall compactness of a protein. In this study, we analyzed the Radius of Gyration (Rg) of both the TLR4 protein and the TLR4-MEV complex, and presented the Rg plot in Fig. 6D. Our findings indicate that the Gyrate value slightly increases upon the combination of MEV with TLR4, suggesting that the compactness of the overall structure of the TLR4 protein is minimally affected by the addition of MEV. Hydrogen bonding (HBond) analysis was conducted to assess interactions between TLR4 and MEV. The HBond plot (Fig. 6E) revealed the formation of numerous hydrogen bonds during the simulation, particularly between key residues in TLR4 and important groups in MEV. By calculating the binding free energy of the MEV-TLR4 complex, we determined a stable binding with a binding free energy (ΔG_MMGBSA_) of −51.13 KCal/Mol, indicating a strong and stable interaction between MEV and TLR4.

### 3.11 Codon optimization and in silico cloning

**Fig. 7:**
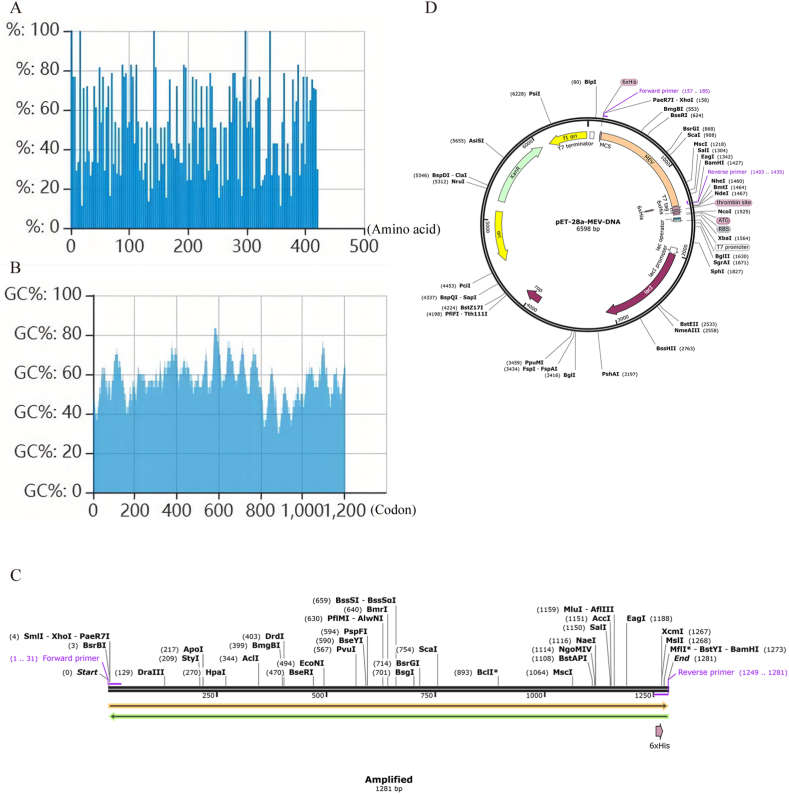
Codon optimization and cloning results of MEV. (A) The CAI after password optimization is 0.80. (B) The GC content after codon optimization is 56.53 %. (C) DNA sequence of MEV after PCR. (D) The orange region is the amplified target gene sequence of MEV inserted into the vector pET28a (+).

In the codon optimization tool ExpOptimizer, the MEV FASTA sequence was reverse translated into a 1263-base DNA sequence. After the MEV DNA sequence was optimized, the codon adaptation index (CAI) was 0.80 and the GC content was 56.53 % (Fig. 7A and B), both values indicate that MEV may be well expressed in E. coli. According to the principles of primer design, we designed a forward primer (5′-ATGCGTGTTCTGTACCTGCTGT-3′) with a length of 22, a Tm value of 61 and a GC content of 50 %, and a reverse primer (5′-ATGATGGTGGTGGTGATGGAACAG-3′) with a length of 24, a Tm value of 60 and a GC content of 50 %, and polymerase chain reaction (PCR) was performed on the DNA sequence of MEV in the software SnapGene7.0.2 (Fig. 7C). The target gene sequence amplified by MEV was inserted into the MCS domain of the vector pET-28a (+) to complete silicon cloning (Fig. 7D).

### 3.12 Simulated agarose gel electrophoresis results

The agarose gel electrophoresis simulation data indicated that the DNA sequence of MEV was 1263 bp, while the MEV DNA sequence post-PCR was 1281 bp. Additionally, the pET-28a (+) DNA sequence measured 5369 bp, and the recombinant plasmid DNA sequence was 6598 bp.

### 3.13 Immune simulation

**Fig. 8:**
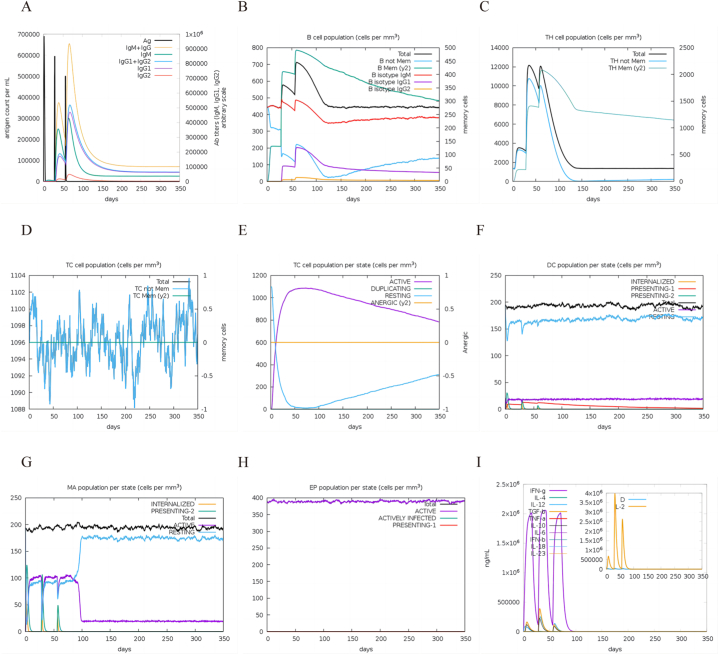
Results of C-ImmSim. (A) Immunoglobulin production following antigen injection. (B) B cell population after three injections. (C) Helper T cell population after three injections. (D) Cytotoxic T cell population after three injections. (E) Number of cytotoxic T cells in each state after three injections. (F) Number of dendritic cells in each state after three injections. (G) Number of macrophages in each state after three injections. (H) EP population in each state after three injections. (I) Simpson index (D) of different levels of cytokines and interleukins and immune responses.

The C-ImmSim server was utilized to simulate the immune response following three injections of MEV. The adaptive immune response is crucial in combatting brucellosis pathogenesis. Fig. 8A illustrates that the body generated elevated levels of specific antibodies after three MEV injections, with IgM and IgG antibody levels peaking post third immunization and gradually declining thereafter. B cells are integral to humoral immunity and immune responses, as depicted in Fig. 8B where total B cells and memory B cells increased with each vaccine dose, peaking after the third immunization before gradually decreasing. Fig. 8C demonstrates a significant increase in total TH cells and non-memory TH cells after the second vaccine stimulation, peaking and then declining. Following the third stimulation, there was a rapid increase in numbers followed by a gradual decrease. Memory TH cells also increased post three MEV stimulations, forming progressively higher peaks and ultimately reaching the highest peak after the third stimulation. Cytotoxic T cells play a role in eliminating *Brucella* through the production of perforin, granzyme B, and other cytotoxic factors. Fig. 8D illustrates that non-memory cytotoxic T cells reached a peak of 1099 cells/mm3 after three stimulations. Following this, Fig. 8E indicates that the number of active cytotoxic T cells in each state peaked after the third stimulation. It is known that dendritic cells (DC) are potent antigen-presenting cells crucial for antigen presentation to T cells. Moving on to Fig. 8F, it is shown that the total number of dendritic cells was maintained at 200 cells/mm3, while the number of active dendritic cells was kept at 20 cells/mm3. Furthermore, present-2 dendritic cells displayed three peaks after three stimulations. Fig. 8G demonstrates that the number of active macrophages was consistently maintained at 100 cells/mm3 from the first to the third injection. However, after 100 days from the initial vaccination, there was a rapid decrease in the number of active macrophages, settling at 20 cells/mm3. Notably, present-2 dendritic cells showed three peaks after three stimulations. Fig. 8H suggests that EP remained relatively stable overall. Lastly, Fig. 8I reveals that vaccination with MEV led to a significant increase in IFN-γ levels. Additionally, following the second immune stimulation, the levels of IFN-γ, IL-12, TGF-β, and other cytokines peaked.

## 4 Discussion

Brucellosis, a zoonotic disease caused by *Brucella* bacteria, can lead to symptoms like abortion and high fever in animals [59]. The current live attenuated Brucella vaccines for animals have limitations including persistent infection in vaccinated animals, potential transmission to humans, risk of virulence reversion, short-lived protection, and potential interference with the diagnosis of brucellosis [60,61]. Human infection with brucellosis presents symptoms such as fever, fatigue, joint pain, and muscle pain [62]. If left untreated, the disease can progress to the chronic phase and may result in complications affecting multiple organs [63].While there are established treatment options for brucellosis involving combination antibiotic therapy, the optimal duration of antibiotic treatment to completely eradicate the bacteria remains unclear. Prolonged treatment can result in adverse effects, reduce treatment adherence [64], and the emergence of antibiotic-resistant Brucella strains, posing significant challenges to treatment.

Prevention is the most effective way to control Brucella, making the development of a vaccine for brucellosis essential. Currently, there is no approved Brucella vaccine for human use, highlighting the need for research into safer and more effective vaccines [60].Developing a new vaccine entails the critical steps of verifying its effectiveness and safety, a process that demands significant manpower, material, and financial resources. Immunoinformatics methods offer a valuable approach for researchers to streamline the experimental burden [65].Therefore, using immunoinformatics approaches to design new kind of vaccines could be a magnificently additive in the way forward of preventing Brucella. Subunit vaccines are crafted using specific pathogen components such as proteins [66]. This category of vaccines includes various types, namely recombinant protein vaccines, epitope vaccines, DNA vaccines, gene marker vaccines, conjugate vaccines, recombinant vector vaccines, nanoparticle vaccines, bacterial shadow vaccines, mRNA vaccines, and others. Notably, epitope vaccines offer distinct advantages when compared to other forms of vaccines [11]. In comparison to traditional vaccines, multi-epitope vaccines offer the advantage of not needing microbial culture, resulting in time and cost savings [67].This study focuses on CTL, HTL epitopes, and B cell antigen epitopes, leading to a broad immune response coverage [68,69]. Additionally, these vaccines sidestep issues like biohazardousness and virulence reversion seen in traditional inactivated or attenuated vaccines. Lastly, the inclusion of highly diverse epitopes allows for recognition by multiple alleles simultaneously, addressing allelic variations within human populations [70].

In this study, we utilized immunoinformatics techniques to design a multi-epitope vaccine targeting *Brucella*. Our findings suggest that Heme transporter BhuA and polysaccharide export protein are promising candidates for constructing such vaccines. Notably, these proteins are associated with *Brucella*'s intracellular infection, bacterial virulence, and drug resistance mechanisms. For instance, Heme transporter BhuA plays a crucial role in utilizing heme as an iron source during *Brucella*'s survival and replication within macrophages [14]. Efficient iron acquisition is vital for bacterial survival within hosts, highlighting the significance of Heme transporter BhuA in *Brucella*'s intracellular persistence.The function of the polysaccharide export protein is to transport polysaccharides. Gram-negative bacteria have the ability to synthesize and export different types of polysaccharides, including capsular polysaccharides (CPS), extracellular polysaccharides (EPS), and lipopolysaccharides (LPS). Research suggests that certain CPS serve as crucial virulence factors [[71], [72], [73]] and protective antigens for bacteria. EPS plays a role in biofilm formation [[74], [75], [76]], contributing to increased antimicrobial resistance. LPS can lead to various severe symptoms such as fever, leukopenia, hypotension, disseminated intravascular coagulation, septic shock, and multiorgan failure. Therefore, inhibiting the transport of polysaccharides by the polysaccharide export protein is of utmost importance. Additionally, an analysis of the antigenicity of Heme transporter BhuA and the polysaccharide export protein revealed predicted antigenicity values of 0.6018 and 0.5500, respectively, both exceeding the 0.4 threshold, indicating strong antigenicity. Previous studies have emphasized the necessity of proteins with good antigenicity for the construction of new MEVs [65]. Furthermore, an assessment of the physical and chemical properties, sensitization, and toxicity of Heme transporter BhuA and the polysaccharide export protein demonstrated that they are stable, hydrophilic, non-allergenic proteins, and are also virulence factors of *Brucella*. Given the current lack of research on constructing MEV based on these two proteins, they have been chosen as candidate proteins for vaccine development.

The multi-epitope vaccine in this study is a subunit vaccine developed using reverse vaccinology methods to predict potential candidate vaccine proteins from the whole genome sequence. Bioinformatics technology is then utilized to identify various antigenic epitopes in silico, ultimately constructing a novel vaccine candidate capable of eliciting the desired immune response. An important aspect of the early stages of Brucella infection is the activation of humoral immunity. However, once Brucella is present intracellularly, the immune response is primarily driven by cellular immunity [59]. CTL plays a role in cellular immunity by specifically targeting and killing pathogen-infected cells and tumor cells through mechanisms like secreting perforin/granzymes and inducing apoptosis pathways. On the other hand, HTL contributes to auxiliary cellular immunity and humoral immunity by producing cytokines that reduce pro-inflammatory responses. B cells play a crucial role in the humoral immune response by carrying out functions such as antigen recognition, antibody production, and immune memory. Predicting T cell epitopes and B cell epitopes to develop a vaccine can lead to comprehensive protection and long-lasting immune responses in vivo. We used different databases and online servers to predict T cell epitopes (CTL epitope and HTL epitope) and B cell epitopes (LBE and CBE) of two proteins to improve the accuracy of prediction. After screening, we identified six CTL dominant epitopes, six HTL dominant epitopes, one conformational B cell dominant epitope, and three linear B cell dominant epitopes with strong immunogenicity, high antigenicity, non-allergenicity, and non-toxicity, all displaying conserved sequences. By utilizing linkers, we connected these epitopes to form a multi-epitope vaccine (MEV). Specifically, GGGS linkers were used for CTL epitopes [68], GPGPG for HTL epitopes, and KK for B cell epitopes. The linker ensures that each epitope can independently trigger an immune response and prevents the generation of new epitopes that may interfere with the immune response induced by the original epitope [77]. To boost the host immune response, a TLR agonist (hBD2) and an auxiliary peptide (PADRE) were connected to the N-terminus of the epitope, improving the overall immunogenicity of the vaccine.

An ideal vaccine should possess not only strong immunogenicity but also specific physical and chemical properties. The physicochemical and immunological properties of MEV were analyzed through various bioinformatics tools. Findings revealed that the molecular weight of MEV is 44.2 kDa. Previous studies suggest that a molecular weight below 110 kDa is ideal for vaccine development [78]. Furthermore, MEV was found to be non-allergenic, non-toxic, non-human homologous, and soluble. These results suggest that MEV has the potential to trigger an immune response without inducing autoimmune reactions.

Bioinformatics software was utilized for molecular docking and molecular dynamics simulations to assess the affinity of MEV towards immune cell receptors, specifically MHC-I, MHC-II, and TLR-4. Analyzing the interactions and intermolecular affinities of these molecules is crucial as they play a key role in initiating an immune response. The findings demonstrate that MEV forms stable complexes with the immune cell receptors MHC-I, MHC-II, and TLR-4 [79].Following simulation with the C-ImmSim server, it has been theoretically demonstrated that the vaccine can lead to optimal cellular and humoral immunity after administration. However, in order to determine the practical effectiveness of the vaccine in preventing brucellosis, additional experiments are essential, despite the anticipated challenges that may arise during the experimental procedures.

## 5 Conclusion

Immunoinformatics methods were utilized to identify multiple dominant epitopes in Heme transporter BhuA and polysaccharide export protein. Adjuvants and auxiliary peptides were then linked to these epitopes to develop a multi-epitope vaccine against *Brucella*. This vaccine exhibits strong immunogenicity, high antigenicity, good stability, strong hydrophilicity, and possesses characteristics such as non-allergenicity, non-toxicity, non-human homology, and solubility. In other words, the multi-epitope vaccine can effectively stimulate a specific immune response without causing harm to the body. Moreover, the multi-epitope vaccine is highly expressed in the E. coli system, making it a promising candidate for a brucellosis vaccine. Nonetheless, further experimental validation is necessary to comprehensively evaluate the potential of these vaccine candidates.

### Ethical statement

Not applicable because there are no animals and patients used in this study.

## Data availability statement

The authors confirm that the data was derived from public domain information: NCBI database (https://www.ncbi.nlm.nih.gov/) and PDB library (https://www.rcsb.org/). The data that support the findings of this study are available in the methods and/or supplementary material of this article. The data that support the findings of this study are available from the corresponding author upon request. There are no restrictions on data availability. If you have any questions, please contact me. Table 3 presents the data sources.

**Table 3:** Data sources.

Type of data	Repositories
Protein sequences	NCBI (https://www.ncbi.nlm.nih.gov/)
Protein structure	PDB library (https://www.rcsb.org/)
Physical and chemical properties of the proteins	ProtParam(https://web.expasy.org/protparam/)
Antigenicity of the protein	VaxiJen 2.0 (http://www.ddg-pharmfac.net/vaxijen/VaxiJen/VaxiJen.html)
Sensitivity analysis of the proteins	AllergenFP v.1.0(https://ddg-pharmfac.net/AllergenFP/)
Predict virulence factor	VirulentPred2.0(https://bioinfo.icgeb.res.in/virulent2/predict.html)
Forecast protein signal peptides	SignalP 6.0 server (https://services.healthtech.dtu.dk/services/SignalP-6.0/) and LiPOP1.0(https://services.healthtech.dtu.dk/services/LipoP-1.0/)
Predict CTL epitopes	IEDB(http://tools.iedb.org/mhci/) and NetCTLpan1 server(https://services.healthtech.dtu.dk/service.php?NetCTLpan-1.1)
Predict HTL epitopes	IEDB(http://tools.iedb.org/mhcii/) and NetMHC-IIpan-4.0((https://services.healthtech.dtu.dk/service.php?NetMHCIIpan-4.0)
Conservation of sequences	NCBI(https://www.ncbi.nlm.nih.gov/Structure/cdd/wrpsb.cgi?INPUT_TYPE=live&SEQUENCE=AIJ88289.1) (https://www.ncbi.nlm.nih.gov/Structure/cdd/wrpsb.cgi?INPUT_TYPE=live&SEQUENCE=UVW32680.1)
Toxicity of sequences	ToxinPred(https://webs.iiitd.edu.in/raghava/toxinpred/design.php)
Predict linear B cell epitopes	SVMtrip(http://sysbio.unl.edu/SVMTriP/prediction.php) IEDB(http://tools.iedb.org/bcell/) ABCpred(https://webs.iiitd.edu.in/raghava/abcpred/ABC_submission.html)
Predict conformational B cell epitopes	IEDB's Ellipro(http://tools.iedb.org/ellipro/)
Human homology	NCBI's BLASTP(https://blast.ncbi.nlm.nih.gov/Blast.cgi?PROGRAM=blastp&PAGE_TYPE=BlastSearch&LINK_LOC=blasthome)
Predict protein solubility	SOLpro(http://scratch. proteomics.ics.uci.edu/)
Predict secondary structure of constructed vaccine	SOPMA(http://www.ibcp.fr/predict.html)
Predict tertiary structure of constructed vaccine	Robetta(https://robetta.bakerlab.org/)
Refine the tertiary structure	Galaxy Refine(https://galaxy.seoklab.org/cgi-bin/submit.cgi?type=REFINE)
Assess the quality of the tertiary structure	Procheck(https://saves.mbi.ucla.edu/)
Molecular docking	HDOCK server(http://hdock.phys.hust.edu.cn/)
Molecular dynamics simulation	Gromacs2022.3 software
Codon adaptation	ExpOptimizer(https://www.novopro.cn/tools/codon-optimization.html)
In silico molecular cloning	SnapGene7.0.2 software
Agarose gel electrophoresis	SnapGene7.0.2 software
Immunosimulation	C-ImmSim(https://kraken.iac.rm.cnr.it/C-IMMSIM/)

## Funding

This work was supported by the 10.13039/501100001809National Natural Science Foundation of China Regional Science Foundation Project (82360394), Youth Science and technology top talent Program (2022TSYCCX0112), Outstanding Youth Science Foundation of Xinjiang Uygur Autonomous Region(2023D01E12), Xinjiang Uygur Autonomous Region science and technology support project (2022E02061) and State Key Laboratory of Pathogenesis, Prevention and Treatment of High Incidence Diseases in Central Asia Fund (SKL-HIDCA2021-JH11).

## CRediT authorship contribution statement

**Kaiyu Shang:** Writing – review & editing, Writing – original draft, Visualization, Software, Methodology, Formal analysis, Data curation, Conceptualization. **Yuejie Zhu:** Writing – review & editing, Methodology, Formal analysis, Conceptualization. **Tingting Tian:** Writing – review & editing, Methodology, Formal analysis. **Huidong Shi:** Writing – review & editing, Methodology, Formal analysis. **Zhengwei Yin:** Software, Resources. **Yueyue He:** Software, Resources. **Juan Shi:** Software, Resources. **Jianbing Ding:** Supervision. **Fengbo Zhang:** Supervision.

## Declaration of competing interest

The authors declare that they have no known competing financial interests or personal relationships that could have appeared to influence the work reported in this paper.
